# A Lethal Progressive Neuroinflammation Disguised as MOGAD Revealing a Final Diagnosis of Griscelli Syndrome

**DOI:** 10.1002/acn3.70470

**Published:** 2026-06-29

**Authors:** Chiara Veredice, Chiara Arpaia, Ilaria Contaldo, Anna Capasso, Donato Rigante

**Affiliations:** ^1^ Pediatric Neurologic Unit Fondazione Policlinico Universitario A. Gemelli IRCCS Rome Italy; ^2^ Department of Life Sciences and Public Health Fondazione Policlinico Universitario A. Gemelli IRCCS Rome Italy; ^3^ Università Cattolica Sacro Cuore Rome Italy

**Keywords:** child, Griscelli syndrome, MOGAD, neuroinflammation, optic neuritis

Dear Editor,

We read with interest the paper by Aduru et al. in which myelin oligodendrocyte glycoprotein antibody‐associated disease (MOGAD) is depicted as the most frequent and relevant cause of first‐time optic neuritis in children [1]. However, the role of MOG‐IgG serum titer changes and oscillations should not be solely used to clarify the nature of neuroinflammatory pictures in children.

A previously healthy 5‐year‐old girl was referred to our Neurologic Unit for fixed right mydriasis and complete right retinal detachment. Neither fever nor right eye pain was ever disclosed. Brain MRI revealed a severe right optic neuritis (Figure 1A), and anti‐MOG antibodies were found positive (1:10) suggesting the diagnosis of MOGAD. Intravenous immunoglobulin (IVIG) at the dose of 2 g/kg monthly were given, but 3 months later a brainstem relapse occurred with vomiting, dysarthria, and ataxia. Brain MRI revealed contrast‐enhancing lesions in supra/infratentorial regions with tonsillar herniation and obstructive hydrocephalus (Figure 1B), requiring third ventriculocisternostomy. High‐dose intravenous methylprednisolone pulses (20 mg/kg/day) were given for 7 days, resulting in partial improvement. Despite all ongoing therapies, the girl relapsed once more after further 3 months, presenting cerebellar ataxia, dysarthria, and right foot drop. Given this refractory‐relapsing MOGAD picture, rituximab (375 mg/m^2^/week) was administered for 4 weeks, but then discontinued after an umpteenth relapse. Mycophenolate mofetil (1000 mg/day) was added to monthly IVIG cycles, leading to a more stable remission lasted 3 years, in which anti‐MOG antibody titers fluctuated, being both positive and negative. Unfortunately, at 9.6 years, the girl developed an abrupt crossed brainstem syndrome with right lower limb hypertonia, flaccid paralysis of the left arm, anarthria, lingual akinesia, and fixed rightward head deviation, with multiple hemorrhagic lesions lined in the thalamus, nucleo‐capsular regions, and pons at a new brain MRI (Figure 1C). Due to this dramatic neurologic deterioration, and rethinking to the peculiar girl's silvery‐gray hair (Figure 1D), a molecular testing panel for hemophagocytic lymphohistiocytosis (HLH) was performed on peripheral blood mononuclear cells. The panel revealed two *RAB27A* variants on chromosome 15q21: c.153+5G>A (a splice site variant which alters pre‐mRNA splicing) and c.514_518del (a pathogenic 5 bp deletion). *RAB27A*‐related disease is an ultrarare condition called Griscelli syndrome type 2 (OMIM #607623) characterized by partial albinism and impaired T cell and natural killer cytotoxic activity with risk of life‐threatening HLH [2]. Dysfunctional Rab27a is coherent with inadequate melanin release within skin and hair, resulting in partial albinism [3]. However, HLH may be virtually absent in some patients with a completely neurologic phenotype, while molecular findings are fully consistent with Griscelli syndrome type 2 [4].

**FIGURE 1:**
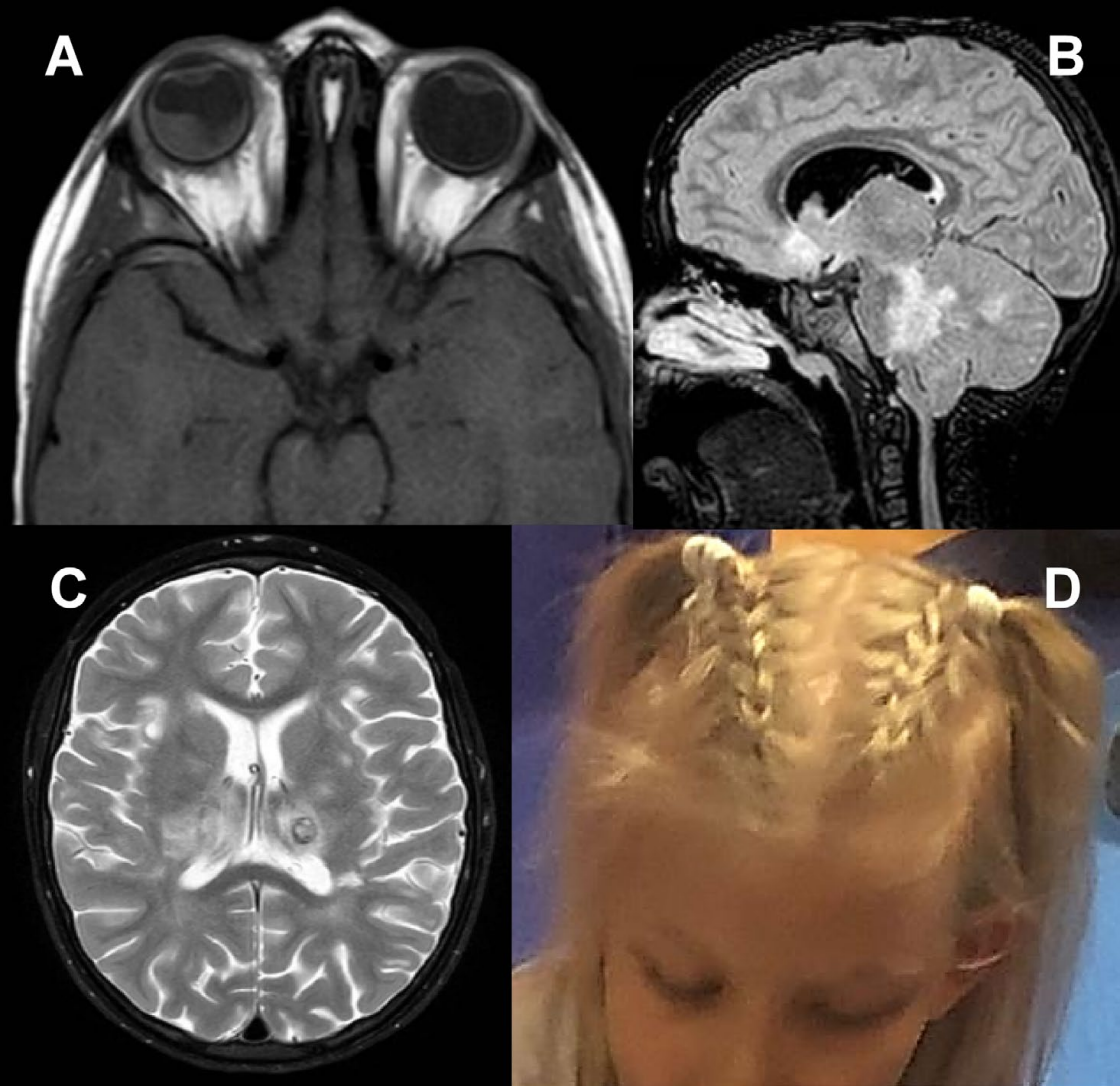
Magnetic resonance imaging revealing: (A) right optic neuritis (hyperintensity on T2); (B) lesions in both supra‐ and infratentorial regions with mass effect causing tonsillar herniation and obstructive hydrocephalus (hyperintense lesions on T2‐FLAIR) 3 months after onset; (C) hemorrhagic lesions in the thalamus, nucleo‐capsular regions, and pons (on T2‐FLAIR) were noted at 9.6 years. (D) Patient's hair and eyebrows with a typical silvery‐gray color.

Ruxolitinib (5 mg b.i.d.) was started soon after diagnosis, and the patient put on list for hematopoietic stem cell transplantation. Unfortunately, a slowly‐progressive multiorgan failure occurred 5 months later, and the girl died at the age of 10.

Rare genetic errors of immunity may coexist with hypopigmentation skin disorders, so health‐care providers should recall that dysregulated immunity may be camouflaged by devastating neuroinflammatory pictures starting with optic neuritis, whereas autoimmune tests like anti‐MOG positivity may taint the true disease identity with positive MOG test not automatically meaning MOGAD.

## Author Contributions

**Chiara Veredice:** conceptualization, investigation, writing and review. **Chiara Arpaia, Ilaria Contaldo, and Anna Capasso:** original draft writing. **Donato Rigante:** conceptualization, review and editing.

## Funding

The authors have nothing to report.

## Ethics Statement

The authors confirm that this manuscript adheres to the ethical policies of the journal, as noted on the journal's author guidelines page, and that it was prepared following the Declaration of Helsinki Ethical Principles for Medical Research Involving Human Subjects.

## Consent

Written informed consent was formally obtained.

## Conflicts of Interest

The authors declare no conflicts of interest.

## Linked Articles

This article is linked to Aduru et al. papers. To view these articles, visit https://doi.org/10.1002/acn3.70471.

## Data Availability

Data sharing not applicable to this article as no datasets were generated or analyzed during the current study.
